# Angiosarcoma: clinical outcomes and prognostic factors, a single-center analysis

**DOI:** 10.1007/s00432-024-05835-x

**Published:** 2024-06-25

**Authors:** Siyer Roohani, Titus Rotermund, Felix Ehret, Tomasz Dziodzio, Armin Jarosch, Frederik Maximilian Schäfer, Anne Flörcken, Silvan Wittenberg, Daniel Zips, David Kaul

**Affiliations:** 1 Department of Radiation Oncology, Charité − Universitätsmedizin Berlin, Corporate Member of Freie Universität Berlin and Humboldt-Universität zu Berlin, Augustenburger Platz 1, 13353 Berlin, Germany; 2 BIH Charité (Junior) Clinician Scientist Program, Berlin Institute of Health at Charité − Universitätsmedizin Berlin, BIH Biomedical Innovation Academy, Charitéplatz 1, 10117 Berlin, Germany; 3 Partner site Berlin, a partnership between DKFZ and Charité, German Cancer Consortium (DKTK), Universitätsmedizin Berlin, Berlin, Germany; 4 Department of Surgery, Charité – Universitätsmedizin Berlin, Corporate Member of Freie Universität Berlin and Humboldt-Universität zu Berlin, Augustenburger Platz 1, 13353 Berlin, Germany; 5 Institute of Pathology, Charité – Universitätsmedizin Berlin, Corporate Member of Freie Universität Berlin and Humboldt-Universität zu Berlin, Charitéplatz 1, 10117 Berlin, Germany; 6 Department of Radiology, Charité – Universitätsmedizin Berlin, Corporate Member of Freie Universität Berlin and Humboldt-Universität Zu Berlin, Berlin, Germany; 7 Department of Hematology, Oncology and Tumor Immunology, Charité − Universitätsmedizin Berlin, Corporate member of Freie Universität Berlin and Humboldt, Universität zu Berlin, Augustenburger Platz 1, 13353 Berlin, Germany; 8 Center for Musculoskeletal Surgery, Charité – Universitätsmedizin Berlin, Corporate Member of Freie Universität Berlin and Humboldt-Universität zu Berlin, Campus Virchow-Klinikum, Augustenburger Platz 1, 13353 Berlin, Germany; 9 Health and Medical University Potsdam, Olympischer Weg 1, 14471 Potsdam, Germany

**Keywords:** Angiosarcoma, Soft tissue sarcoma, Outcomes, Risk factors, Surgery, Radiotherapy, Chemotherapy

## Abstract

**Purpose:**

This study sought to investigate oncological outcomes and prognostic factors for patients with angiosarcomas (AS).

**Methods:**

This single-center, retrospective cohort study, analyzed histopathologically confirmed AS cases. Primarily diagnosed, locally recurrent and metastatic AS were included. Overall survival (OS), local control (LC) and local progression-free survival (LPFS) were assessed by Kaplan-Meier estimator. Multivariable Cox regression analysis was performed to detect factors associated with OS and LPFS.

**Results:**

In total, 118 patients with a median follow-up of 6.6 months were included. The majority presented with localized disease (62.7%), followed by metastatic (31.4%) and locally recurrent (5.9%) disease. Seventy-four patients (62.7%) received surgery, of which 29 (39.2%) were treated with surgery only, 38 (51.4%) with surgery and perioperative radiotherapy or chemotherapy, and 7 (9.4%) with surgery, perioperative radiotherapy and chemotherapy. Multivariable Cox regression of OS showed a significant association with age per year (hazard ratio (HR): 1.03, *p* = 0.044) and metastatic disease at presentation (hazard ratio: 3.24, *p* = 0.015). For LPFS, age per year (HR: 1.04, *p* = 0.008), locally recurrent disease at presentation (HR: 5.32, *p* = 0.013), and metastatic disease at presentation (HR: 4.06, *p* = 0.009) had significant associations. Tumor size, epithelioid components, margin status, and perioperative RT and/or CTX were not significantly associated with OS or LPFS.

**Conclusion:**

Older age and metastatic disease at initial presentation status were negatively associated with OS and LPFS. Innovative and collaborative effort is warranted to overcome the epidemiologic challenges of AS by collecting multi-institutional datasets, characterizing AS molecularly and identifying new perioperative therapies to improve patient outcomes.

## Introduction

Angiosarcomas (AS) are rare malignant vascular tumors of endothelial cell origin [1, 2]. They account for 2–4% of soft tissue sarcoma (STS), have a male predominance, and most frequently affect patients aged 60–70 years [3, 4]. For the majority of AS, the etiology is unknown [1, 2]. However, established risk factors are chronic lymphedema of any origin (Stewart-Treves syndrome), exposure to ionizing radiation (typically years after radiotherapy (RT)), chemicals (vinyl chloride, arsenic, radium), vascular malformations (arteriovenous fistulas, pre-existing hemangioma), and rare genetic syndromes (neurofibromatosis, Mafucci syndrome) [1, 2, 5, 6]. Moreover, prior trauma or surgery as well as long-term exposure to implanted foreign material are associated with AS development [1, 2]. More than half of all AS arise from the skin followed by soft tissues of the lower extremities, retroperitoneum, trunk wall and head and neck region [1].

Within the group of STS, AS have one of the highest risks of distant metastatic spread (most commonly to the lung) and, for this reason, among others, one of the worst prognoses [7–9]. Another remarkable feature of AS among all STS subtypes is the higher tendency to lymph node and central nervous system metastatic spread [9, 10]. Adverse prognostic factors in AS are older age, retroperitoneal location, large tumor size, and positive surgical margins [1, 8, 9, 11]. For localized cutaneous AS, wide surgical resection has repeatedly demonstrated its essential role in improving oncological outcomes [12, 13]. A number of retrospective studies suggest that the addition of postoperative RT improves overall survival (OS) and local control (LC) in localized cutaneous AS [12, 14, 15]. For metastatic AS, taxane-based chemotherapy (CTX) regimens are recommended [10, 16, 17]. This single-center retrospective study sought to investigate clinical outcomes, prognostic factors and the role of perioperative combination therapies for the management of AS.

## Methods

In this retrospective, single-center cohort study adult patients with histopathologically confirmed AS who received treatment at our institution between 2009 and 2023 were included. Patients presenting with primarily diagnosed, locally recurrent or metastatic AS were included. Patients below 18 years of age were excluded. Data on the patient characteristics, imaging, pathology, surgical, oncological, RT treatment characteristics, and oncological outcome data were reviewed. Oncological endpoints of interest included OS, LC and local progression-free survival (LPFS). OS was defined as the time from primary diagnosis to death by any cause. LC was defined as an unchanged or decreased AS volume after surgical excision or last RT treatment (if not resected) or last CTX cycle (if not resected or irradiated) assessed by a board-certified radiologist on follow-up imaging with magnetic resonance imaging (MRI) or computed tomography (CT). LPFS was defined as the time from surgical excision or last RT treatment (if not resected), or last CTX cycle (if not resected or irradiated) to histopathological or radiographic evidence of AS volume increase or local recurrence on clinical or radiographic follow-up examinations or death by any cause. Clinical follow-up was calculated from the date of initial therapy until the last clinical visit. Radiographic follow-up was calculated from the day of initial therapy until the last available MRI or CT. Patients were censored at the last available follow-up if no local recurrence or death were observed. Data on survival status was obtained from the tumor registry of the Charité Comprehensive Cancer Center. In the event of a registered death, the information was integrated into the OS and LPFS analysis.

For descriptive statistics, ranges, medians, interquartile ranges, and means for continuous variables were used. OS and LPFS were assessed using the Kaplan-Meier estimator. Multivariable Cox regression was performed to analyze factors associated with OS and LPFS. A p-value of ≤ 0.05 was considered statistically significant. The proportional hazards assumption was tested and fulfilled for all analyzed endpoints using Schoenfeld residuals. Statistical analysis and figure design was performed with STATA MP 16.0 (StataCorp, College Station, TX, USA). The study was approved by the institutional review board (EA1/072/23).

## Results

### Patient and treatment characteristics

Patient and treatment characteristics are summarized in Table 1. A total of 118 patients were included. At primary diagnosis, the median age was 67 years (range 18–95), with a slight male predominance (54.2% males, 45.8% females). The most common primary tumor sites were skin (22.0%) and soft tissue (22.0%), followed by breast (11.0%) and liver (11.0%). The majority of patients presented with a localized primary diagnosis of AS (74, 62.7%). Thirty-seven (31.4%) patients had primary diagnoses of metastatic disease at initial presentation, and 7 (5.9%) patients presented with a locally recurrent AS. In 37 patients with metastatic disease, 68 metastatic sites were detected. The most common metastatic site was bone (27.9%), followed by lymph nodes (22.1%), lungs (20.6%), other sites (17.6%), and liver (11.8%). Primary tumor size was smaller or equal to 5 cm in 42 (35.6% ) patients, while 39 (33.1%) were larger than 5 cm. In 7 patients (5.9%), primary tumors were multifocal and in 3 (2.5%) patients, tumors were diffusely infiltrating. Information on tumor size was not available in 27 patients (22.9%). Four patients (3.4%) had chronic lymphedema-associated AS (Stewart-Treves syndrome) and 36 tumors (30.5%) carried epithelioid features. In 13 patients (11.0%) with radiation-induced AS, the median time from radiation exposure to primary diagnosis in the same location was 7.7 years (range: 3.3–26.0). Eleven out of the 13 (84.6%) radiation-induced AS cases were located in the breast. Most patients underwent surgery (62.7%). A total of 28 patients (23.7%) were treated with RT, of which the majority was given postoperatively (75.0%). Four patients (14.3%) were treated with RT only. CTX was given to 50 patients (42.4%). Of these 50 patients, 25 (50%) were treated with paclitaxel, 19 (38%) with anthracycline-based regimens, and 6 (12%) received other chemotherapy regimens. Within this group, the CTX was administered perioperatively to 29 patients (58.0%), while 20 patients (40.0%) were treated with CTX only and 1 (2.0%) received radiochemotherapy only. Seventy-four patients (62.7%) received surgery, of which 29 (39.2%) were treated with surgery only, 38 (51.4%) with surgery and perioperative RT or CTX and 7 (9.4%) with surgery, perioperative RT and CTX.

**Table 1:** Patient and treatment characteristics

	All *N* = 118	
Characteristics	N	%
Median age in years (range)	67 (18–95)	
Sex		
Female	54	45.8
Male	64	54.2
Site		
Skin	26	22.0
Soft Tissue	26	22.0
Breast	13	11.0
Liver	13	11.0
Bone	9	7.7
Heart	7	5.9
Unknown	4	3.5
Other*	20	16.9
Presentation status		
Localized, primary diagnosis	74	62.7
Localized, recurrent disease	7	5.9
Metastatic, primary diagnosis	37	31.4
Metastatic site	68	100.0
Bone	19	27.9
Lymph node	15	22.1
Lungs	14	20.6
Liver	8	11.8
Others	12	17.6
Size		
≤ 5 cm	42	35.6
> 5 cm	39	33.1
Multifocal	7	5.9
Diffuse	3	2.5
N/A	27	22.9
Chronic lymphedema-associated angiosarcoma (Stewart-Treves syndrome)	4	3.4
Radiation-induced angiosarcoma	13	11.0
Median time in years between radiation and primary diagnosis angiosarcoma (range)	7.7 (3.3–26.0)	
Epithelioid component	36	30.5
Surgery		
Yes	74	62.7
No	39	33.1
N/A	5	4.2
Radiotherapy	28	23.7
Preoperative RT	2	7.1
Postoperative RT	21	75.0
Radiotherapy only	4	14.3
Concurrent radiochemotherapy only	1	3.6
Median dose per fraction (range)	2.0 (1.0–5.0)	
Median total dose (range)	59.0 (15.0–66.0)	
Chemotherapy	50	42.4
Preoperative CTX	12	24.0
Postoperative CTX	17	34.0
CTX only	20	40.0
Concurrent radiochemotherapy only	1	2.0
Combination therapies		
Surgery	74	62.7
Surgery only	29	39.2
Surgery with perioperative RT or CTX	38	51.4
Surgery with perioperative RT and CTX	7	9.4

^*^Other sites included: Lungs, esophagus, tonsils, uterus, adrenal glands, kidneys, ovaries, paranasal sinuses, lymph nodes, parotid glands, thyroid gland and spleen.

Abbreviations: cm = centimeter; CTX = Chemotherapy; N/A = Not available; RT = Radiotherapy

### Oncological outcomes

**Fig. 1:**
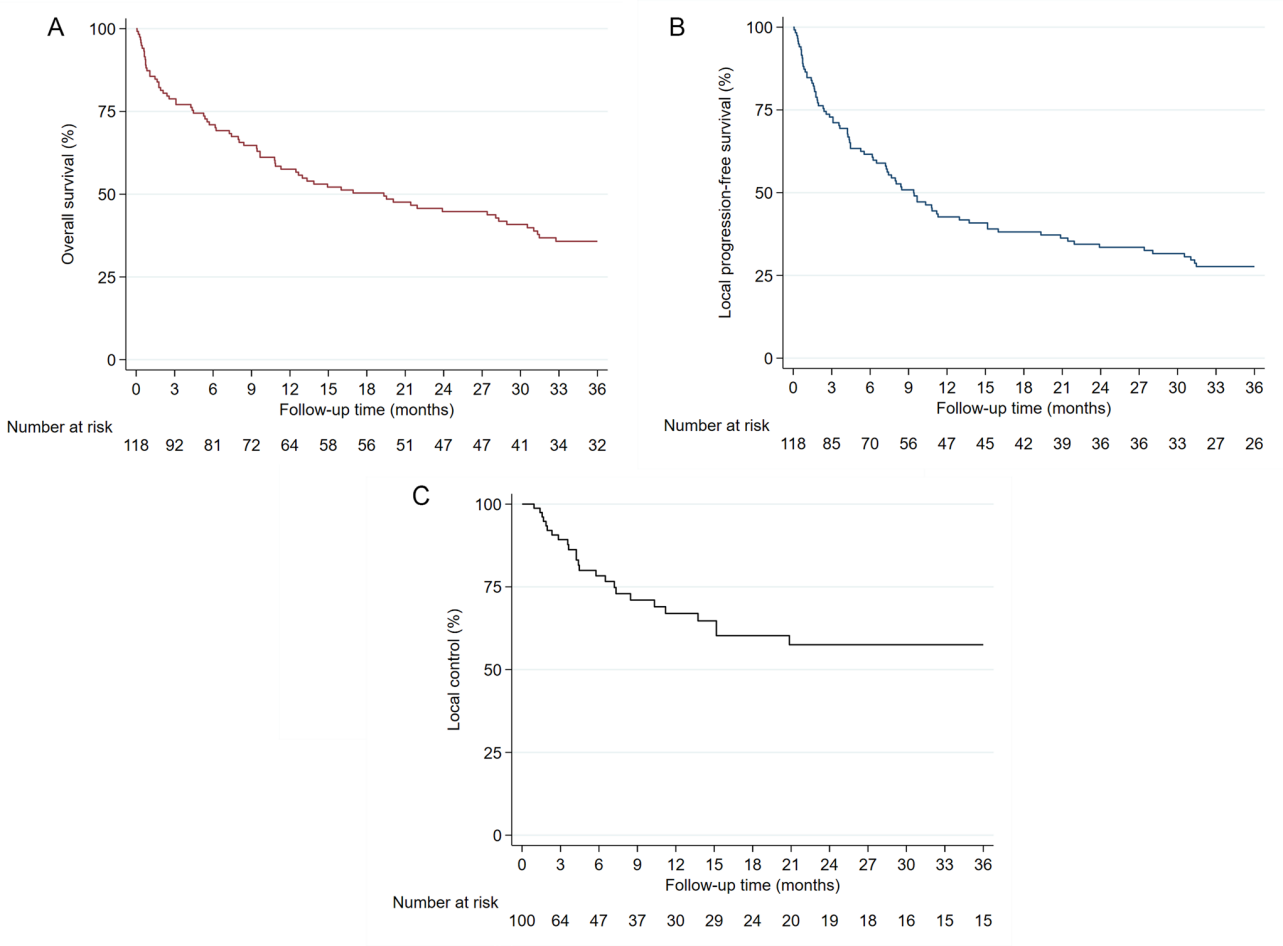
Overall survival (**A**), local progression-free survival (**B**) and local control (**C**) in the entire study cohort

Oncological outcomes are summarized in Table 2. The median clinical follow-up was 6.6 months, and the median radiographic follow-up was 7.9 months. In 67 patients with available resection margin status, the majority (76.1%) had clear surgical margins, while 16 patients (13.6%) had positive surgical margins. Of these, 8 (6.8%) of which were microscopically positive, and 8 (6.8%) were macroscopically positive. In the entire study cohort, the median OS was 19.3 months (95% confidence interval: 10.8–28.9 months). The 3-month, 6-month, 1-year, and 2-year OS rates were 78.8%, 71.0%, 57.5%, and 44.7%, respectively (Fig. 1A). The median LPFS was 9.4 months (95% confidence interval: 6.5–13.7 months). The 3-month, 6-month, 1-year, and 2-year LPFS rates were 72.8%, 61.5%, 42.6% and 33.5%, respectively (Fig. 1B). Data on local disease control were available for 100 out of 118 patients. The median LC was not reached. The 3-month, 6-month, 1-year, and 2-year LC rates were 89.6%, 78.8%, 67.2% and 57.7%, respectively.

**Table 2:** Oncological outcomes

	Median	Mean	IQR		Range
Clinical follow-up, months	6.6	16.9	19.8		0.1-123.2
Radiographic follow-up, months	7.3	19.3	21.5		0.1-123.2
	N = 118				
	*N*		*%*		
Resection margin	67		56.8		
R0	51		76.1		
R1	8		6.8		
R2	8		6.8		
N/A	51		-		
	Median (months)	3 months (%)	6 months (%)	1 year (%)	2 years (%)
Overall survival	19.3 (CI: 10.8–28.9)	78.8	71.0	57.5	44.7
Local progression-free survival	9.4 (CI: 6.5–13.7)	72.8	61.5	42.6	33.5
Local control	Not reached	89.6	78.8	67.2	57.7

Abbreviations: CI = 95% confidence interval; IQR = Interquartile range; N/A = Not available

### Prognostic factors

In the multivariable Cox regression analysis, age per year (hazard ratio: 1.03, *p* = 0.044), and metastatic disease at presentation (hazard ratio: 3.24, *p* = 0.015) were significantly associated with OS (Table 3). Tumor size, epithelioid components, perioperative RT and/or CTX, and locally recurrent disease at presentation were not significantly associated with OS. For LPFS, age per year (hazard ratio: 1.04, *p* = 0.008), locally recurrent disease at presentation (hazard ratio: 5.32, *p* = 0.013), and metastatic disease at presentation (hazard ratio: 4.06, *p* = 0.009) had significant associations (Table 4). Tumor size, epithelioid components, margin status, and perioperative RT and/or CTX were not significantly associated with LPFS.

**Table 3:** Multivariable Cox proportional hazards model for overall survival

Variable	Hazard ratio	Confidence interval (95%)	*p*-value
Age (in years)	1.03	1.00–1.05	0.044
Size			
≤ 5 cm	Reference		
> 5 cm	1.10	0.52–2.35	0.798
Epithelioid features			
No epithelioid components	Reference		
Epithelioid components	0.65	0.25–1.68	0.371
Unknown	1.45	0.59–3.56	0.424
Combination therapies			
Surgery	Reference		
Surgery with perioperative RT or CTX	0.88	0.37–2.06	0.763
Surgery with perioperative RT and CTX	1.49	0.41–5.35	0.543
Presentation status			
Localized primary diagnosis	Reference		
Locally recurrent	1.89	0.37–9.64	0.443
Metastatic primary diagnosis	3.24	1.26–8.36	0.015

Abbreviations: CTX = Chemotherapy; RT = Radiotherapy

**Table 4:** Multivariable Cox proportional hazards model for local progression-free survival

Variable	Hazard ratio	Confidence interval (95%)	*p*-value
Age	1.04	1.01–1.06	0.008
Size			
≤ 5 cm	Reference		
> 5 cm	1.04	0.50–2.18	0.913
Epithelioid features			
No epithelioid components	Reference		
Epithelioid components	0.44	0.17–1.11	0.080
Unknown	0.77	0.33–1.82	0.556
Margin status			
Negative	Reference		
Positive	0.94	0.36–2.46	0.893
Combination therapies			
Surgery	Reference		
Surgery with perioperative RT or CTX	1.43	0.63–3.24	0.394
Surgery with perioperative RT and CTX	0.96	0.27–3.38	0.944
Presentation status			
Localized primary diagnosis	Reference		
Locally recurrent	5.33	1.41–20.07	0.013
Metastatic primary diagnosis	4.06	1.42–11.56	0.009

Abbreviations: CTX = Chemotherapy; RT = Radiotherapy

### Subgroup analyses

**Fig. 2:**
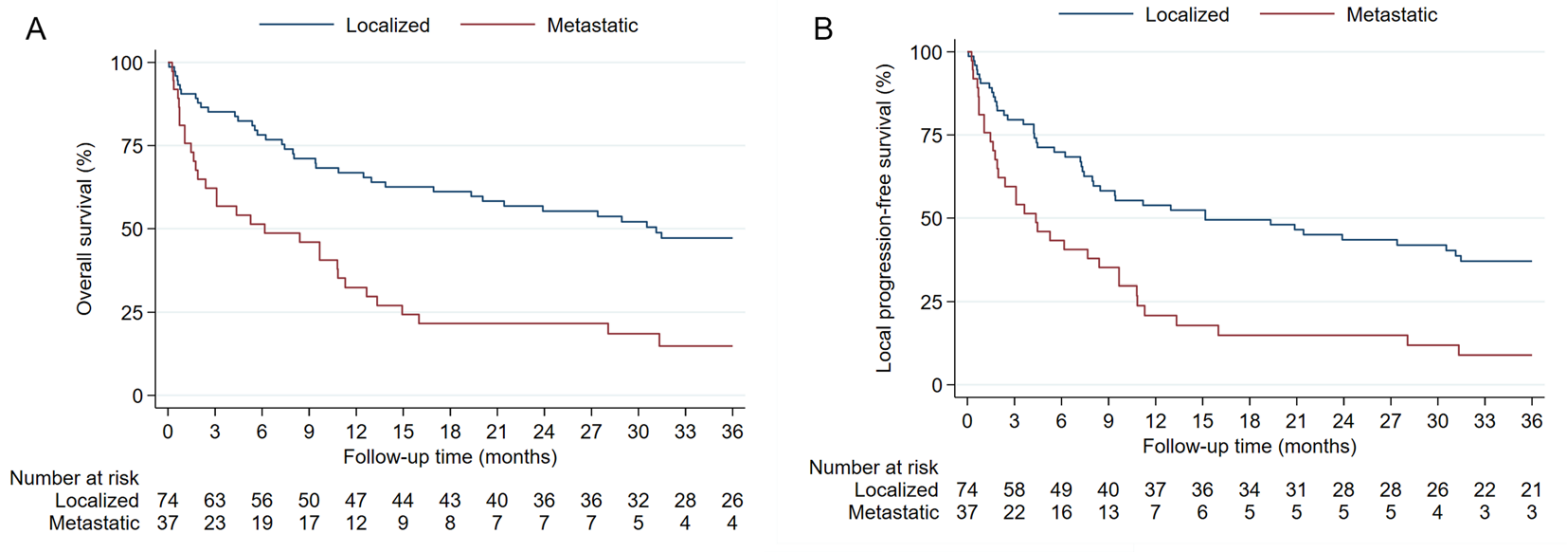
Overall survival (**A**) and local progression-free survival (**B**) in patients with localized and metastatic disease at presentation

The OS and LPFS Kaplan-Meier estimates stratified by localized vs. metastatic disease at initial presentation are displayed in Fig. 2. In patients with localized disease, the median OS of 31.1 months was strikingly longer than the median OS of 6.2 months in patients presenting with metastatic disease (Fig. 2A). Similarly, the median LPFS of 15.1 months in localized disease at presentation was longer than the 4.4 months in patients with metastatic disease (Fig. 2B).

## Discussion

In this single-center retrospective cohort study, we report data on 118 AS patients. Despite intensive treatment, the overall prognosis of patients is limited. Age and metastatic disease at presentation were significant adverse prognostic factors for OS, and local disease progression after therapy.

The median age of 67 years at initial presentation is within the range described in large analyses, ranging from 62 to 73 years of age [11, 18, 19]. Age, as an important clinical factor associated with OS and LPFS in our cohort, was repeatedly identified in other studies as well [8, 9, 18]. This association is not very surprising, as, depending on the tumor location, the curative treatment requires intensive multimodal therapies. Moreover, AS are prone to local and distant recurrences requiring repeated demanding therapies, which may not be amenable to patients of a certain age without risking considerable therapy-limiting side effects [9]. As expected, distant metastasis at initial presentation was also associated with poor OS and LPFS in our cohort. This finding confirms previous studies and may account for the short OS in our cohort, as a comparably large proportion (31.4%) of patients initially presented with metastatic disease [16, 19–22]. Additionally, the median OS of 19.3 months and 1 year OS of 57.5% reported herein are comparable to previous cohort studies on localized and metastatic AS [9, 11].

The combination of surgery and RT was associated with improved OS, LC and disease-specific survival in a retrospective single-center cohort study on 70 localized AS cases from the MD Anderson Cancer Center by Guadagnolo et al. [12]. In our study, combination therapies (surgery with RT or surgery with CTX vs. surgery alone) were not significantly associated with OS or LPFS. These findings can be explained by the inclusion of metastatic cases in our study and the smaller number of analyzable cases who were treated with combination therapies. For the LC endpoint, the low number of local recurrences in our cohort impeded multivariable Cox regression analyses. Current European guidelines recommend the combination of wide surgical excision and preoperative CTX or RT for localized AS [10]. Thus, larger cohort studies are needed to further investigate the role of perioperative treatments in addition to surgery for localized AS. In our cohort, locally recurrent disease at presentation was significantly associated with LPFS and not significantly associated with OS. Lahat et al. did not detect a significant associations between this factor and local disease recurrence or disease-specific survival in a single-center study on 222 AS patients (11). Although presenting with locally recurrent disease does suggest an aggressive tumor biology and could indicate a higher risk of further local disease recurrences as seen in our cohort, the small subgroup of 7 patients in our cohort may be biased and results should be interpreted in light of this limitation.

Tumor size of > 5 cm is repeatedly described as another adverse prognostic factor for OS and disease-specific survival in AS patients [12, 19]. We did not identify a significant association with OS or LPFS. This may be on the one hand due to missing values and on the other hand due to difficulties assessing tumor sizes in AS. In comparison to other sarcomas, AS are known to diffusely infiltrate the skin or cause multifocal disease which often impede correct and precise measurements of tumor size [2]. Epithelioid features have been reported to correlate with increased rates of local disease recurrence and mortality [8, 11, 23]. In our study cohort, we were not able to confirm this observation and even found a tendency towards reduced risk of a LPFS event with epithelioid features. However, the informative value is limited due to the small number of cases.

Secondary AS due to radiation exposure (stochastic effect of RT) are not the most common radiation-induced sarcomas. However, they do represent the most common subtype of secondary sarcoma after irradiation of breast tissue [24]. In line with this, 11 out of 13 radiation-induced AS in our cohort were breast AS. Moreover, the median time of 7.7 years from previous radiation exposure to the development of radiation-induced AS in our study confirms previous studies reporting median times of 6.5-8 years [11, 18].

Painter et al. undertook an innovative and inclusive approach to overcome the rarity of AS and gain valuable insights into its mutational landscape in the Angiosarcoma Project [25]. In this project, the authors generated an open and easy to use online platform for AS patients and relatives in the US and Canada to consent for uploading medical records and send in saliva and blood samples, which will be verified and analyzed with whole exome sequencing. Within 18 months, 227 patients consented and a total of 47 samples from 36 patients were used for genomic analysis. The authors found striking differences in tumor mutational burden, and the pattern of molecular alterations among the different locations and subtypes of AS. For instance, AS of the head and neck region not only had a significantly higher tumor mutational burden compared to AS of other regions but also a mutational signature commonly found in damage from ultraviolet light indicating solar radiation to be a reproducible etiology for AS in sun-exposed areas [25]. A similar, ultraviolet light-induced mutational pattern was also described in melanoma and correlates with response to immune checkpoint inhibitors [26–28]. Intriguingly, about 21% of all AS samples in the Angiosarcoma Project carried PIK3CA mutations which are targetable with FDA-approved drugs according to the OncoKB precision oncology database [25, 29]. Although the sample size is yet too small to draw firm conclusions for clinical practice, the project still has taken an important, innovative and patient-inclusive step towards overcoming AS as rare cancers and unravelling the group of AS to identify targetable mutations for clinical trials. Future results on this project are awaited with great interest.

### Future perspective

A number of different treatment schedules for AS are investigated in clinical trials that are currently recruiting or have been completed with pending results. Based on preclinical data showing high ß-adrenergic receptor expression in AS and hemangioma and the clinical benefits of beta-blockers for infantile hemangioma, a drug-repurposing trial using propranolol in addition to standard therapy for AS has recently reached completion [30–32]. The results are pending. The aforementioned similarity in mutational patterns of head and neck AS to melanomas and the proven efficacy of immune checkpoint inhibition in melanomas and in multiple other cancer types with high tumor mutational burden lay a valuable foundation for future studies (25, 27, 28, 33). Several smaller studies reported durable responses with checkpoint inhibitors in the treatment of AS (34–36). An ongoing multi-center Scandinavian phase 2 study is examining this rationale using pembrolizumab combined with the beta-blocker propranolol for advanced AS or undifferentiated pleomorphic sarcoma to study the effect on progression-free survival (37). Another prospective, multi-center, single-arm trial examines the overall response rate after 6 months of checkpoint inhibition with cemiplimab in cutaneous and secondary AS (38). A different approach is investigating synergistic effects of paclitaxel with RT for localized cutaneous AS in an ongoing clinical trial treating patients with 6 weekly cycles of paclitaxel followed by paclitaxel with RT (39). The phase 3 TAPPAS trial compared the non-VEGF pathway anti-angiogenic monoclonal antibody carotuximab combined with pazopanib to pazopanib alone in patients with advanced AS (40). The study did not reach its primary endpoint of superior progression-free survival by the combination therapy over pazopanib alone (40). Despite its negative results, the study demonstrated how multi-center efforts can overcome the challenges in rare cancers by achieving a remarkable number of 114 recruited AS patients within two years of recruitment. Future multicenter joint projects are necessary to identify molecular targets and test new combination therapies to improve outcomes for AS patients.

### Limitations

The present study carries the limitations inherent to retrospective, single-center studies. The total number of reported LC events was too small to apply multivariable Cox regression analyses for this endpoint. Importantly, our study also included patients presenting with locally recurrent or metastatic disease which may skew the observed survival times. Missing values in certain subdomains (epithelioid features, tumor size, primary tumor location) also limit the informative value. However, it also reflect the clinical reality, as assessing epithelioid features in a rare STS subtype may not be a standard procedure and measuring tumor size in often diffusely infiltrating, multifocal AS is challenging or impossible.

## Conclusions

AS are rare and aggressive subtypes of STS with dismal prognoses. Older age and metastatic disease at initial presentation status were adverse prognostic factors for local recurrence and OS in our study cohort. Innovative and collaborative effort is warranted to overcome the challenges of AS through collecting multi-institutional datasets, further characterizing AS molecularly and identifying new perioperative therapies to improve patient outcomes.

## Data Availability

Data available on request from the corresponding author.

## Abbreviations

AS: Angiosarcoma
CT: Computed tomography
CTX: Chemotherapy
FNCLCC: Fédération Nationale des Centres de Lutte Contre le Cancer
IQR: Interquartile range
LC: Local control
LPFS: Local progression-free survival
MRI: Magnetic resonance imaging
N/A: Not available
OS: Overall survival
RT: Radiotherapy
STS: Soft tissue sarcoma
